# Prevalence, Determinants and Outcomes of Intravenous-to-Oral Switch of Antibiotic Therapy in Hospitalized Adults With Bloodstream Infection: Secondary Analysis of a Multicenter Cohort

**DOI:** 10.1093/ofid/ofag666

**Published:** 2026-10-19

**Authors:** Lea A Nikolai, Beryl P Gladstone, Sarah V Walker, Jörg J Vehreschild, Kristina Schmauder, Simone Eisenbeis, Alexander Mischnik, Evelyn Kramme, Christine Geffers, Siegbert Rieg, Trinad Chakraborty, Maria J G T Vehreschild, Harald Seifert, Jan Rupp, Silke Peter, Winfried V Kern, Evelina Tacconelli, Daniel Hornuss, Siri Göpel, for the DZIF BLOOMY study group, Elke Lemke, Norbert Thoma, Solvy Wolke, Can Imirzalioglu, Susanne Herold, Nicole Tewes, Moritz Fritzenwanker, Annika Classen, David Tobys, Paul Higgins, Nadja Käding, Benjamin Gebel, Lisa Höltig, Katharina Nagel, Larissa Künstle, Elisabeth Stoll, Ariane Dinkelacker, Gabriele Peyerl-Hoffmann, Georg Häcker, Heike Spitznagel, Sara Olawumi-Hutter

**Affiliations:** Department of Internal Medicine I, University Hospital Tübingen, Tübingen, Germany; Department of Internal Medicine I, University Hospital Tübingen, Tübingen, Germany; DZIF–Clinical Research Unit, Infectious Diseases, Department of Internal Medicine I, University Hospital Tübingen, Tübingen, Germany; DZIF German Center for Infection Research, Braunschweig, Germany; DZIF German Center for Infection Research, Braunschweig, Germany; Institute for Medical Microbiology, Immunology, and Hygiene, Faculty of Medicine and University Hospital Cologne, University of Cologne, Cologne, Germany; Goethe University Frankfurt, Faculty of Medicine, Institute for Digital Medicine and Clinical Data Sciences, Frankfurt/Main, Germany; DZIF German Center for Infection Research, Braunschweig, Germany; Institute of Medical Microbiology and Hygiene, University Hospital Tübingen, Tübingen, Germany; Excellence Cluster 2124 “Controlling Microbes to Fight Infections” (CMFI), University of Tübingen, Tübingen, Germany; Department of Internal Medicine I, University Hospital Tübingen, Tübingen, Germany; DZIF German Center for Infection Research, Braunschweig, Germany; DZIF German Center for Infection Research, Braunschweig, Germany; Division of Infectious Diseases, Department of Medicine II, Medical Center—University of Freiburg, Faculty of Medicine, University of Freiburg, Freiburg, Germany; Infectious Disease Clinic, University of Lübeck and University Hospital Schleswig-Holstein/Campus Lübeck, Lübeck, Germany; DZIF German Center for Infection Research, Braunschweig, Germany; Infectious Disease Clinic, University of Lübeck and University Hospital Schleswig-Holstein/Campus Lübeck, Lübeck, Germany; DZIF German Center for Infection Research, Braunschweig, Germany; Institute of Hygiene and Environmental Medicine, Charité – Universitätsmedizin Berlin, Berlin, Germany; DZIF German Center for Infection Research, Braunschweig, Germany; Division of Infectious Diseases, Department of Medicine II, Medical Center—University of Freiburg, Faculty of Medicine, University of Freiburg, Freiburg, Germany; DZIF German Center for Infection Research, Braunschweig, Germany; Institute of Medical Microbiology, Justus Liebig University Giessen, Giessen, Germany; DZIF German Center for Infection Research, Braunschweig, Germany; Department of Internal Medicine, Infectious Diseases, Goethe University Frankfurt, University Hospital Frankfurt, Frankfurt/Main, Germany; Department I of Internal Medicine, University Hospital of Cologne, Cologne, Germany; DZIF German Center for Infection Research, Braunschweig, Germany; Institute for Medical Microbiology, Immunology, and Hygiene, Faculty of Medicine and University Hospital Cologne, University of Cologne, Cologne, Germany; Institute of Translational Research, CECAD Cluster of Excellence, University of Cologne, Cologne, Germany; DZIF German Center for Infection Research, Braunschweig, Germany; Infectious Disease Clinic, University of Lübeck and University Hospital Schleswig-Holstein/Campus Lübeck, Lübeck, Germany; Institute of Medical Microbiology, University Hospital Schleswig-Holstein/Campus Kiel & Lübeck, Kiel, Germany; DZIF German Center for Infection Research, Braunschweig, Germany; Institute of Medical Microbiology and Hygiene, University Hospital Tübingen, Tübingen, Germany; Division of Infectious Diseases, Department of Medicine II, Medical Center—University of Freiburg, Faculty of Medicine, University of Freiburg, Freiburg, Germany; Department of Internal Medicine I, University Hospital Tübingen, Tübingen, Germany; DZIF German Center for Infection Research, Braunschweig, Germany; Division of Infectious Diseases, Department of Diagnostic and Public Health, University of Verona, Policlinico GB Rossi, Verona, Italy; DZIF German Center for Infection Research, Braunschweig, Germany; Division of Infectious Diseases, Department of Medicine II, Medical Center—University of Freiburg, Faculty of Medicine, University of Freiburg, Freiburg, Germany; Department of Internal Medicine I, University Hospital Tübingen, Tübingen, Germany; DZIF–Clinical Research Unit, Infectious Diseases, Department of Internal Medicine I, University Hospital Tübingen, Tübingen, Germany; DZIF German Center for Infection Research, Braunschweig, Germany

**Keywords:** antimicrobial stewardship, bloodstream infection, intravenous-to-oral switch, oral antibiotics, rational antibiotic use

## Abstract

**Background:**

Intravenous-to-oral switch (IVOS) is considered safe in selected patients with bloodstream infection (BSI) and provides operational advantages. Still, it is underused in many clinical settings. We evaluated IVOS patterns, associated factors, and clinical outcomes in patients with BSI.

**Methods:**

Secondary data were analyzed from 2 prospective, multicenter cohort studies recruiting patients with BSI. IVOS was assessed on day 3 and 7 after positive blood culture. Eligibility for IVOS was based on infectious focus, clinical safe-switch criteria, and antimicrobial susceptibility. Risk factors for non-IVOS were studied using multivariable logistic regression.

**Results:**

Among 3824 patients, 2770 were included in the secondary analysis, and 742 (19.4%) were eligible and evaluable for IVOS. By day 7, 240 patients (32.3%) underwent IVOS. Switch occurred on the day of discharge in 107 patients (44.6%), and 1 day before discharge in 46 (19.2%). The most commonly used oral agents were ciprofloxacin (75 patients [31.3%]), sultamicillin (56 [23.3%]), and cefuroxime axetil (27 [11.3%]). Barriers to IVOS on multivariable analysis included diabetes (odds ratio, 0.57 [95% confidence interval, .38–.88]; *P* = .01), chronic kidney disease (0.64 [.41–.99]; *P* = .048), Pitt bacteremia score ≥2 on day 3 (0.43 [.19–.97]; *P* = .04), and septic shock before day 7 (0.31 [.11–.87]; *P* = .03), while urogenital focus (2.22 [1.50–3.28]; *P* < .001) and 2 study centers were positively associated with IVOS. IVOS was associated with shorter length of stay (7 vs 12 days; *P* < .001) and a lower 28-day mortality rate.

**Conclusions:**

In clinically stable patients with BSI, IVOS was infrequently implemented. Barriers to IVOS should be addressed as part of antimicrobial stewardship programs.

## Teaser

In a tertiary care setting, intravenous-to-oral antibiotic switch (IVOS) was underused in clinically stable patients with bloodstream infection. Implementation barriers included comorbid conditions, more severe disease, and nonurogenital focus. Patients with IVOS had shorter hospital stays and a lower mortality rate.

Intravenous-to-oral switch (IVOS) is considered an important component of appropriate antibiotic therapy [1]. IVOS is generally recommended in clinically stable patients after 72 hours, including in selected cases of bloodstream infection (BSI) [2, 3], making it a key component of antimicrobial stewardship (AMS) programs [4, 5].

IVOS offers several advantages, including shorter length of hospital stay (LOS), reduced risk of catheter-associated infections, and cost savings [6–10]. Concerns regarding the safety of IVOS have been addressed in several clinical settings, with studies showing noninferiority of oral therapy when clinical criteria are met, including in BSI [6, 11]. However, the role of IVOS in *Staphylococcus aureus* BSI remains debated, with recent evidence suggesting noninferiority in low-risk cases [12].

Nonetheless, IVOS remains underused, and addressing both perceived and actual barriers to IVOS implementation is an important challenge [13–15]. In this context, there is a need to better understand current IVOS practices in the hospital setting.

BLOOMY and BLOOMY-PREDICT are 2 prospective, multicenter cohort studies recruiting patients with microbiologically confirmed BSI in German university hospitals [16]. Using these cohorts, we conducted a secondary analysis to investigate IVOS patterns in hospitalized adults with BSI caused by ESKAPE pathogens, excluding *S aureus* BSI as the role of oral antibiotic therapy in this setting remains controversial. The primary objectives were to determine the frequency of IVOS and to identify factors associated with non-IVOS. The secondary objective was to assess the association between IVOS and clinical outcomes.

## METHODS

### Study Cohort

Clinical data were prospectively collected in 6 German university hospitals for a derivation cohort (ClinicalTrials.gov NCT03578835) and in 5 of these hospitals for a validation cohort. The 2 studies included adults (aged ≥18 years) with ≥1 blood culture positive for *S aureus*, *Enterococcus* spp, *Escherichia coli*, *Klebsiella* spp, *Enterobacter* spp, *Pseudomonas aeruginosa*, or *Acinetobacter baumannii.* Laboratory values and vital parameters were collected on day 0 (day of index blood culture collection), day 3, and weekly from day 7 onward. Antibiotic treatment data were collected daily.

Between February 2017 and December 2020, a total of 3824 patients were included in the study [16]. Databases were built in REDCap (Research Electronic Data Capture) tools [17, 18]. During the study period, basic AMS measures were in place in all study centers, including infectious diseases (ID) consultation services on demand. In 3 study centers, ID consultation routinely included recommendations regarding IVOS, and general IVOS guidelines were available in 3 of the 6 study centers. Further details on data collection and processing are given in Supplementary Table 1 and the Supplementary Methods, and study definitions are provided in the Supplementary Methods.

For the current secondary analysis, we defined the following inclusion criteria: first BSI episode during the study period, survival and hospital stay until day 3, monomicrobial BSI, and antibiotic therapy given on day 3 covering the pathogen based on antimicrobial susceptibility testing (AST). Patients were further excluded if no antibiotic was given or antibiotic therapy was insufficiently documented. Based on this cohort, IVOS and antibiotic de-escalation were separately assessed as quality indicators using distinct eligibility criteria.

### Patient Consent Statement

Ethical approval was given by the Ethics Commission of the University Hospital Tübingen (nos. 765/2016BO1 and 584/2019BO1), and all participating centers obtained approval from local ethics committees. Written consent for study participation and publication of data was obtained, and patient information was anonymized to the extent possible. All study procedures were in accordance with the ethical standards of the Helsinki Declaration.

### Definition of IVOS

IVOS was defined as switch to an oral antibiotic in patients initially receiving intravenous antibiotic therapy. If a patient received combination therapy, all prescribed antibiotics had to be given orally to be considered switched. IVOS had to be performed by day 7 at the latest, and IVOS by day 3 was considered early IVOS.

### Eligibility for IVOS

IVOS eligibility was determined in a stepwise manner based on patient and infection characteristics, clinical stability, and microbiological factors. In accordance with the evidence current at the time of recruitment, IVOS eligibility was based on absence of all of the following: immunosuppression, including fever in neutropenia; positive follow-up blood culture obtained until day 7; BSI with *S aureus*; complicating infectious focus (endocarditis, central nervous system infection, pleural empyema, bone and joint infection); and foreign body–associated infection involving difficult-to-remove devices (transurethral urinary catheters, peripheral venous catheters, and central venous catheters were considered easily removable, and patients with these devices were thus eligible for IVOS) [19].

To assess clinical stability, patients had to fulfill safe-switch criteria, which were met when all of the following applied: treatment outside the intensive care unit on the evaluation days; no parenteral nutrition; decreased or unchanged C-reactive protein value on day 3 compared with day 0 *or* a decreased or unchanged leukocyte count on day 3 compared with day 0 and ≥4000/µL; a Pitt bacteremia score (PBS) <4 on day 3; and, if the PBS was 2 or 3, a decreased or unchanged PBS on day 3 compared with day 0 [3, 20]. On day 7, the same criteria for safe switch were used, except for PBS (which was not collected on day 7), and laboratory values were compared between day 3 and day 7.

### Assessment of Switch Options

If patients fulfilling safe-switch criteria were not yet switched by day 7, we assessed whether an oral option had been available to avoid misclassification as non-IVOS. An oral option was considered to be present if AST showed antibiotics with sufficient oral bioavailability as susceptible or if patients received an intravenous antibiotic with an oral equivalent with sufficient bioavailability (criteria as listed in the Supplementary Methods) and no allergies against the available options were known. If no oral option was identified, patients were excluded from analysis.

### Statistical Methods

The primary outcome of interest was the rate of IVOS among patients considered to be eligible and with an IVOS option. The only patients evaluated were those started on intravenous therapy and receiving antibiotics until day 3 for early IVOS or day 7 for late IVOS. IVOS rates were studied among subgroups based on patient characteristics and clinical, infection-specific, and pathogen-specific covariates. Associations with categorical variables were tested using the χ^2^ statistic and associations with continuous variables using the nonparametric equality-of-medians test. Differences were considered statistically significant at *P* < .05.

Multivariable mixed effect logistic regression analyses were performed to study the factors associated with IVOS and the association of IVOS with the 28-day mortality rate. A backward stepwise method was used on covariates with a univariate association of *P* ≤ .2 to build the final model. As specific study centers had a strong association with IVOS, not explained by any studied covariate, study center was used as a covariate in the regression model for factors associated with IVOS to study potential center-level prescribing practices, along with a center-level random effect to account for clustering.

Results are presented as odds ratios along with 95% confidence intervals, and data analyses were performed using Stata/SE software, version 18 [21]. Results were reported in accordance with the STROBE statement (Supplementary Table 2).

## RESULTS

Of 3824 patients included in the original study cohort, 2770 (72.4%) were included in the secondary analysis (Figure 1). After application of eligibility criteria, including safe-switch criteria, 821 of 3824 patients (21.5%) remained. Among these, no oral alternative to intravenous therapy was available in 79 (9.6%). The final study cohort therefore included 742 of 3824 patients (19.4%) with clinically and microbiologically feasible IVOS. Characteristics of included patients are presented in Table 1 and Supplementary Tables 3 and 4.

**Figure 1.**
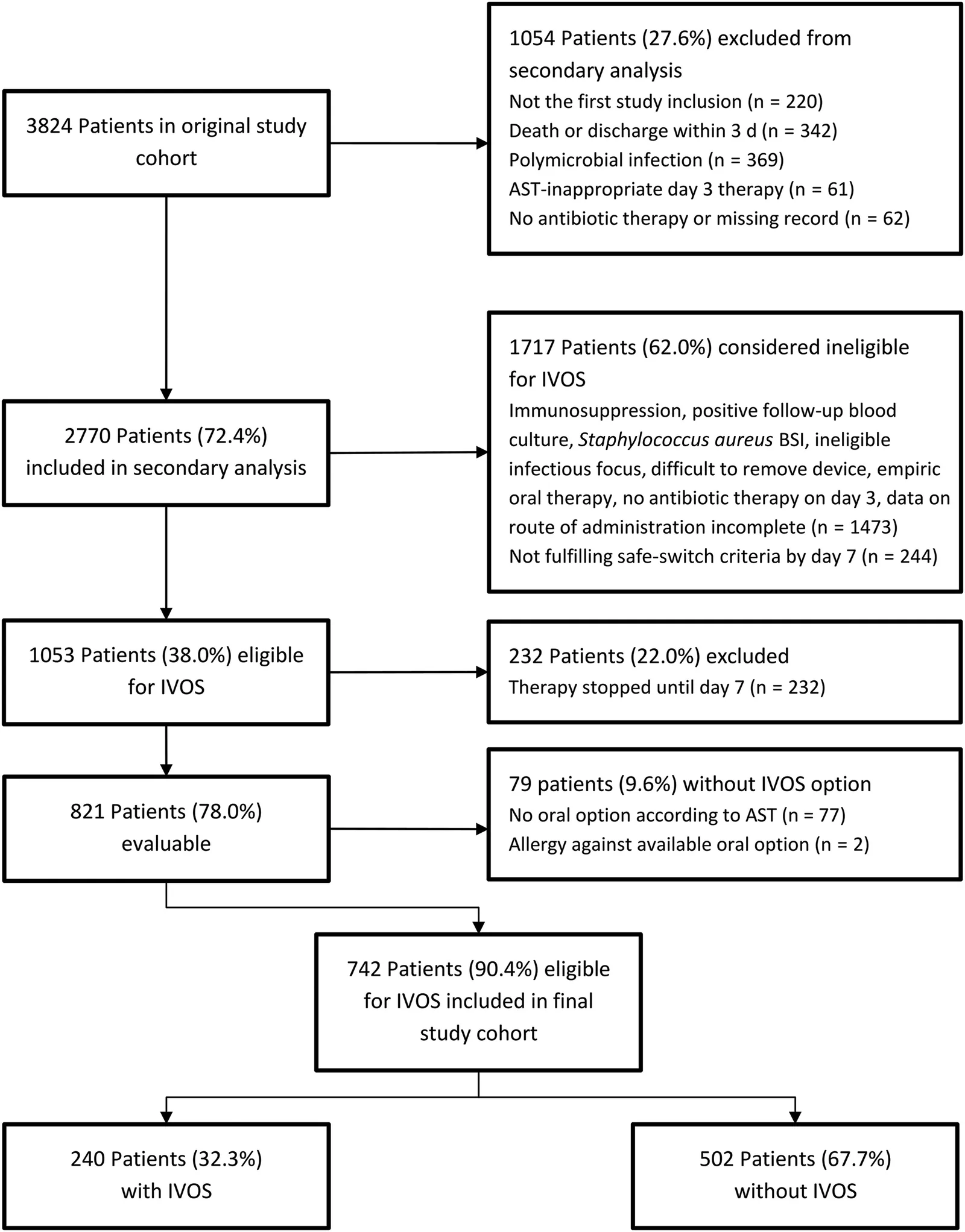
Flowchart for the evaluation of intravenous-to-oral switch (IVOS). Abbreviations: AST, antimicrobial susceptibility testing; BSI, bloodstream infection.

**Table 1.** Characteristics of Included Patients

Variable	Patients, No. (%)^a^	
	Included in Secondary Analysis (n = 2770)	Included in Final Study Cohort (Eligible and Evaluable for IVOS) (n = 742)
Female sex	1004 (36.5)	310 (42.1)
Age, y		
≤40	156 (5.6)	29 (3.9)
41–60	645 (23.3)	145 (19.5)
61–80	1494 (53.9)	401 (54.0)
>80	475 (17.1)	167 (22.5)
CCI ≥3	960 (35.3)	257 (35.1)
Cancer	1204 (43.5)	285 (38.5)
Moderate-to-severe renal disease	796 (29.2)	191 (26.2)
Diabetes	752 (27.1)	206 (27.8)
CHF	656 (24.6)	142 (19.7)
Nursing home residence	79 (2.9)	36 (4.9)
ICU admission in past 3 mo	332 (13.0)	57 (8.3)
In ICU on d 0	690 (24.9)	91 (12.3)
PBS on d 0		
<2	1211 (68.0)	352 (73.2)
≥2	569 (32.0)	129 (26.8)
PBS on d 3		
<2	1937 (77.9)	624 (90.8)
≥2	551 (22.1)	63 (9.2)
Type of acquisition		
Community acquired	513 (18.5)	227 (30.6)
Healthcare associated	1074 (38.8)	326 (43.9)
Hospital acquired	1183 (42.7)	189 (25.5)
MDR infection in past 3 mo	285 (10.3)	37 (5.0)
Focus of infection		
Abdominal	438 (15.8)	183 (24.7)
CNS	10 (0.4)	…
Urogenital	732 (26.4)	375 (50.5)
Respiratory	253 (9.1)	46 (6.2)
Cardiovascular	422 (15.2)	16 (2.2)
Skin and soft tissue	156 (5.6)	15 (2.0)
Bone and joints	73 (2.6)	…
Multiple foci	176 (6.4)	21 (2.8)
Unknown focus	510 (18.4)	86 (11.6)
Foreign body–associated infection	517 (18.7)	43 (5.8)
BSI pathogen		
*Acinetobacter baumannii*	6 (0.2)	0 (0.0)
*Enterobacter* (*Klebsiella*) *aerogenes*	23 (0.8)	8 (1.1)
*Enterobacter cloacae* complex	108 (3.9)	40 (5.4)
*Enterococcus faecalis*	174 (6.3)	64 (8.6)
*Enterococcus faecium*	232 (8.4)	49 (6.6)
*Escherichia coli*	1039 (37.5)	445 (60.0)
*Klebsiella oxytoca*	67 (2.4)	25 (3.4)
*Klebsiella pneumoniae*	225 (8.1)	86 (11.6)
*Pseudomonas aeruginosa*	128 (4.6)	22 (3.0)
*Staphylococcus aureus*	761 (27.5)	…
Other	7 (0.3)	3 (0.4)
MDR pathogen	281 (10.1)	40 (5.4)
Adverse events (after d 7)		
Septic shock	61 (2.3)	5 (0.7)
AKI	90 (3.4)	9 (1.3)
Mortality rate		
In-hospital	555 (20.0)	45 (6.1)
28-d	476 (17.2)	55 (7.4)
6-mo	704 (32.6)	126 (22.2)
LOS, median (IQR) (after positive index blood culture), d	14 (9–23)	10 (8–15)
Study center		
Center 1	483 (17.4)	88 (11.9)
Center 2	898 (32.4)	247 (33.3)
Center 3	61 (2.2)	11 (1.5)
Center 4	234 (8.4)	42 (5.7)
Center 5	594 (21.4)	192 (25.9)
Center 6	500 (18.1)	162 (21.8)

Abbreviations: AKI, acute kidney injury; BSI, bloodstream infection; CCI, Charlson comorbidity index; CHF, congestive heart failure; CNS, central nervous system; ICU, intensive care unit; IQR, interquartile range; IVOS, intravenous-to-oral switch; LOS, length of hospital stay; MDR, multidrug resistance; PBS, Pitt bacteremia score.

^a^Data represent no. (%) of patients unless otherwise specified. Denominators varied due to missing values. The totals for variables were as follows for the secondary analysis and the final cohort, respectively: sex, 2749 and 737; CCI, 2722 and 732; cancer 2767 and 740; moderate-to-severe chronic kidney disease, 2730 and 730; CHF, 2662 and 719; ICU admission in the past 3 months, 2551 and 683; PBS at day 0, 1780 and 481; PBS at day 3, 2488 and 687; septic shock, 2628 and 731; AKI, 2626 and 711; and 6-month mortality rate, 2160 and 567.

### Prevalence of IVOS

Of the 742 patients with an option for IVOS, 240 (32.3%) had been switched to oral therapy by day 7 (Figure 1). In 46 of these 240 patients (19.2%), IVOS was performed early. Conversely, 502 patients (67.7%) continued to receive intravenous therapy on day 7. The available oral options in non-IVOS patients based on AST were ciprofloxacin in 360 of 414 gram-negative cases (87.0%) and cotrimoxazole in 326 of 397 gram-negative non-*Pseudomonas* cases (82.1%) according to AST. For *Enterococcus* spp, AST showed susceptibility to ampicillin in 47 of 88 cases (53.4%) and to linezolid in 83 of 88 (94.3%). In addition, 4 of the 502 patients received intravenous antibiotics on day 7 despite the availability of oral equivalents: ampicillin-sulbactam in 3 cases, and linezolid in 1 case. IVOS rates showed no significant variation (*P* = .41) over the study period, with 35.7% of patients (87 of 244) being switched in 2017, 32.2% (86 of 267) in 2018, 34.4% (11 of 32) in 2019, and 28.1% (56 of 199) in 2020.

### Patient Characteristics Associated With IVOS

When comparing patients with IVOS with patients who remained on intravenous treatment, we found a lower burden of several comorbid conditions, lower day 3 PBS, and more frequent urogenital focus among the switched patients (Table 2). In addition, switched patients tended to be younger and more often had community-acquired or healthcare-associated infection. IVOS rates differed significantly between participating hospitals, a difference that was independent of the distribution of pathogens and foci (Supplementary Tables 5 and 6).

**Table 2.** Characteristics of Patients With Switched Versus Nonswitched Therapy (n = 742)

Variable	Patients, No. (%)		*P* Value^b^
	Switched (n = 240 [32.3%])^a^	Nonswitched (n = 502 [67.7%])^a^	
Female sex	107 (34.5)	203 (65.5)	.27
Age, y			
≤40	15 (51.7)	14 (48.3)	.06
41–60	42 (29.0)	103 (71.0)	
61–80	136 (33.9)	265 (66.1)	
>80	47 (28.1)	120 (71.9)	
CCI ≥3	61 (23.7)	196 (76.3)	<.001
Cancer	76 (26.7)	209 (73.3)	.008
Moderate-to-severe renal disease	50 (26.2)	141 (73.8)	.02
Diabetes	50 (24.3)	156 (75.7)	.004
CHF	33 (23.2)	109 (76.7)	.007
Nursing home residence	11 (30.6)	25 (69.4)	.81
ICU admission in past 3 mo	11 (19.3)	46 (80.7)	.02
In ICU on d 0	14 (15.4)	77 (84.6)	<.001
PBS on d 3			
<2	213 (34.1)	411 (65.9)	.003
≥2	10 (15.9)	53 (84.1)	
Type of acquisition			
Community acquired	81 (35.7)	146 (64.3)	.001
Healthcare associated	118 (36.2)	208 (63.8)	
Hospital acquired	41 (21.7)	148 (78.3)	
MDR infection in past 3 mo	10 (27.0)	27 (73.0)	.48
Focus of infection			
Abdominal	45 (24.6)	138 (75.4)	<.001
Urogenital	154 (41.1)	221 (58.9)	
Respiratory	5 (10.9)	41 (89.1)	
Cardiovascular	3 (18.8)	13 (81.3)	
Skin and soft tissue	4 (26.7)	11 (73.3)	
Multiple foci	3 (14.3)	18 (85.7)	
Unknown focus	26 (30.2)	60 (69.8)	
Foreign body–associated infection	8 (18.6)	35 (81.4)	.047
BSI pathogen			
*Enterobacter* (*Klebsiella*) *aerogenes*	1 (12.5)	7 (87.5)	.004
*Enterobacter cloacae* complex	14 (35.0)	26 (65.0)	
*Enterococcus faecalis*	21 (32.8)	43 (67.2)	
*Enterococcus faecium*	4 (8.2)	45 (91.8)	
*Escherichia coli*	164 (36.9)	281 (63.1)	
*Klebsiella oxytoca*	6 (24.0)	19 (76.0)	
*Klebsiella pneumoniae*	25 (29.1)	61 (70.9)	
*Pseudomonas aeruginosa*	5 (22.7)	17 (77.3)	
Other	0 (0.0)	3 (100.0)	
MDR pathogen	7 (17.5)	33 (82.5)	.04
Study center			
Center 1	16 (18.2)	72 (81.8)	<.001
Center 2	51 (20.6)	196 (79.4)	
Center 3	4 (36.4)	7 (63.6)	
Center 4	12 (28.6)	30 (71.4)	
Center 5	65 (33.9)	127 (66.1)	
Center 6	92 (56.8)	70 (43.2)	

Abbreviations: BSI, bloodstream infection; CCI, Charlson comorbidity index; CHF, congestive heart failure; ICU, intensive care unit; MDR, multidrug resistance; PBS, Pitt bacteremia score.

^a^Denominators varied due to missing values. The totals for variables were as follows for the switched and nonswitched patient groups, respectively: sex, 238 and 499; CCI, 236 and 496; cancer, 240 and 500; moderate-to-severe renal disease, 239 and 491; CHF, 236 and 483; ICU admission in the past 3 months; 226 and 457; and PBS on day 3, 223 and 464.

^b^ *P* values based on χ^2^ test.

Relevant patient characteristics associated with IVOS were integrated into a multivariable model (Supplementary Table 7). In the final model, septic shock until day 7, day 3 PBS ≥2, moderate-to-severe chronic kidney disease, and diabetes were independently associated with a higher probability of non-IVOS. Higher IVOS rates were found in BSI secondary to urogenital infection and with study centers 5 and 6 (Table 3).

**Table 3.** Final Multivariable Mixed Effect Logistic Regression Model of Factors Associated With Intravenous-to-Oral Switch Among Patients With an Option to Switch

Variable	Patients, No. (n = 667)^a^	Multivariable Final Model	
		Adjusted OR (95% CI)	*P* Value
Age, y			
≤40	28	1 (Reference)	
41–60	133	0.54 (.21–1.35)	.19
61–80	359	0.93 (.39–2.19)	.87
>80	147	0.57 (.23–1.43)	.23
Moderate-to-severe renal disease			
No	495	1 (Reference)	
Yes	172	0.64 (.41–.99)	.048^b^
Diabetes			
No	475	1 (Reference)	
Yes	192	0.57 (.38–.88)	.01^b^
CHF			
No	522	1 (Reference)	
Yes	129	0.64 (.38–1.06)	.08
Unknown	16	0.50 (.14–1.87)	.31
PBS on d 3			
<2	605	1 (Reference)	
≥2	62	0.43 (.19–.97)	.04^b^
Septic shock before d 7			
No	629	1 (Reference)	
Yes	38	0.31 (.11–.87)	.03^b^
Focus of infection			
All other foci	293	1 (Reference)	
Respiratory	37	0.40 (0.13–1.25)	.11
Urogenital	337	2.22 (1.50–3.28)	<.001^b^
MDR pathogen			
No	632	1 (Reference)	
Yes	35	0.35 (.13–1.00)	.050
Study center			
Center 1	73	1 (Reference)	
Center 2	236	0.92 (.45–1.90)	.83
Center 3	9	2.12 (.43–10.45)	.36
Center 4	40	1.84 (.70–4.85)	.22
Center 5	151	2.19 (1.06–4.51)	.03^b^
Center 6	158	6.35 (3.06–13.17)	<.001^b^

Abbreviations: CHF, congestive heart failure; CI, confidence interval; MDR, multidrug resistance; OR, odds ratio; PBS, Pitt bacteremia score.

^a^Data were available for all variables included in the final model for 667 of the 742 patients.

^b^Significant at *P* < .05.

We further compared characteristics of patients with IVOS by day 3 and those with IVOS by day 7 (Supplementary Table 8). Early IVOS was more common in younger patients as well as in study center 2, but the differences were not significant. Other factors, including burden of comorbidity, site of acquisition, BSI focus, and the infecting pathogen, were not associated with the time point of IVOS. The median length of antibiotic therapy was shorter among patients with early IVOS than among those with late IVOS (10 vs 11 days, respectively; *P* = .01).

### Patterns of Antibiotic Use

For oral therapy, the most used antibiotics were ciprofloxacin (75 patients [31.3%]), sultamicillin (56 [23.3%]), and cefuroxime axetil (27 [11.3%]). The most frequently used antibiotic class was fluoroquinolones (88 patients [36.7%]), followed by aminopenicillin–β-lactamase inhibitors (74 [30.8%]) and second-generation cephalosporins (27 [11.3%]). When comparing empiric antibiotics between those with and without IVOS, we found that initial antibiotic therapy was similar in the 2 groups. Piperacillin-tazobactam was the most commonly administered antibiotic in both groups (in 73 of 240 [30.4%] and 139 of 502 [27.7%], respectively). For patient with IVOS, this was followed by ampicillin-sulbactam (52 [21.7%]) and ceftriaxone (38 [15.8%]), and in those without IVOS it was followed by ceftriaxone (83 [16.5%]) and ampicillin-sulbactam (69 [13.7%]). In the non-IVOS group, there was little change in antibiotic use until day 7, contrasting with the evident shifts in prescribing patterns seen among those with IVOS (Supplementary Figures 1 and 2). Supplementary Tables 9–14 provide an overview of the antibiotic regimens used in the cohort.

Most antibiotics after IVOS were from a different drug class when compared with the initial agents. However, patients receiving empiric monotherapy with an agent that has a suitable oral equivalent were switched significantly more often than those receiving other empiric regimens (70 of 170 [41.2%] vs 170 of 572 [29.7%], respectively; *P* = .005). Indeed, 45.2% of those empirically treated with a fluoroquinolone and 43.0% of those treated with an empiric aminopenicillin–ß-lactamase inhibitor-combination were switched, compared with the overall IVOS rate of 32.3%.

### Outcomes Associated With IVOS

The LOS was significantly shorter in patients with IVOS than in those in whom an opportunity for IVOS was missed (Table 4). IVOS occurred on the day of discharge in 107 of 240 patients (44.6%), 1 day before in 46 of 240 (19.2%), and 2 days before in 23 of 240 (9.6%). The remaining 64 patients (26.7%) stayed in hospital for >2 days after IVOS.

**Table 4.** Length of Antibiotic Therapy and Patient Outcomes With Switched Versus Nonswitched Therapy (n = 742)

Variable	Patients With Switched Therapy (n = 240 [32.3%])^a^	Patients With Nonswitched Therapy (n = 502 [67.7%])^a^	*P* Value^b^
Total length of antibiotic therapy, median (IQR), d	11 (9–13)	11 (8–15)	.69
LOS, median (IQR), d (after positive blood culture)	7 (6–8.5)	12 (10–18)	<.001
Adverse events, no. (%) (after d 7)			
Septic shock	0 (0.0)	5 (1.0)	.12
AKI	0 (0.0)	9 (1.9)	.03
Mortality rate, no. (%)			
In-hospital	0 (0.0)	45 (9.0)	<.001
28-d	2 (0.8)	53 (10.7)	<.001
6-mo	22 (11.6)	104 (27.5)	<.001

Abbreviations: AKI, acute kidney injury; IQR, interquartile range; LOS, length of hospital stay.

^a^Denominators varied due to missing values. The totals for variables were as follows for the switched and nonswitched patient groups, respectively: septic shock, 237 and 494; acute kidney injury, 235 and 476, and 6-month mortality rate, 189 and 378.

^b^ *P* values based on Wilcoxon rank sum test for continuous variables and χ^2^ test for categorial variables.

Both short- and long-term mortality rates were significantly lower among patients with IVOS. To explore this relationship further, an explanatory multivariable analysis of factors associated with the 28-day mortality rate was performed. In the final multivariable model integrating cancer diagnosis, metastasized solid tumor, day 3 PBS, focus of BSI, and site of acquisition, IVOS remained independently associated with reduced mortality rate (Supplementary Table 15).

## DISCUSSION

In this secondary analysis of a prospective, multicenter BSI cohort study, we found that fewer than one-third of patients with an IVOS option were switched to oral antibiotics by day 7. Factors independently associated with IVOS included urogenital focus, treatment in specific study centers, and more favorable clinical condition, as measured by lower day 3 PBS, absence of septic shock, and fewer comorbid conditions. IVOS was further associated with a significant reduction in LOS and a lower 28-day mortality rate.

In US-based studies investigating IVOS in patients with BSI at a similar time point, switch rates varied between 40.5% and 44.0% [22–24]. However, these studies focused on gram-negative pathogens, and their inclusion criteria were heterogeneous, with Engers et al [22] considering nearly all patients with BSI eligible for IVOS, while Tamma et al [23] evaluated only those with source control, functioning oral intake, and confirmed clinical stability. Another US study, investigating IVOS in enterococcal BSI, found a switch rate of 33.6%, higher than the 22% of patients with enterococcal BSI who were switched in our study [25].

The low IVOS rates in our cohort raise the question of which obstacles to switching were present. Our findings corroborate those of previous BSI studies, which also found higher PBS [22–24, 26, 27] and higher burden of comorbidity [22, 27] to be associated with non-IVOS. In our study, diabetes and chronic kidney disease were identified as independent factors associated with non-IVOS on multivariable analysis, possibly because both conditions serve as surrogate markers of frailty or perceived infection complexity, which were otherwise not sufficiently captured in our database. Clinicians might also hesitate to use some common oral antibiotic options in these patients. For example, the risk of adverse drug events associated with fluoroquinolones is increased in both patients with diabetes and chronic kidney disease [28]. Furthermore, the use of cotrimoxazole in patients with chronic kidney disease requires particular caution, as cotrimoxazole exposure in this population has been found to be associated with an increased risk of acute kidney injury and hyperkalemia [29]. In addition, particularly high IVOS rates in BSI secondary to urogenital infection were previously reported [22, 23, 26, 27] and were confirmed in our analysis. The perception of this infectious focus as clinically less complex could have contributed to this finding, along with the suitability of many commonly used oral antibiotics for urogenital infections.

Nonetheless, the identified barriers alone are not enough to justify the hesitancy concerning IVOS in our cohort of clinically stable patients, especially given the growing evidence of its safety. Previous studies have shown that reservations about IVOS persist, with misconceptions regarding the pharmacological safety of oral antibiotics compared with intravenous drugs remaining common among physicians [14, 15]. Compared with US or French physicians, German ID specialists have been found to be particularly reluctant [30, 31], recommending IVOS less often and at a later time point [31]. In addition, the antibiotic use patterns found in our cohort show underuse of favorable oral agents. Cefuroxime axetil was the third-most-used oral antibiotic despite its lower bioavailability, while antibiotics with superior pharmacological properties, such as cotrimoxazole or amoxicillin-clavulanic acid, remained infrequently used.

Previous studies have suggested that the choice of empiric antibiotics could influence later IVOS [32], with IVOS being more frequent when its implementation does not require switch to a different antibiotic agent or even a different antibiotic class [33]. Indeed, in our study IVOS was performed more often when empiric monotherapy with an orally available antibiotic was chosen. We further observed that in center 6, which had the highest rate of IVOS, ampicillin-sulbactam was more commonly used for empiric therapy than in other centers (23.5% vs 13.1%, respectively; *P* = .001). Local customs of empiric therapy choice may therefore also explain part of the variations in IVOS rates found between the centers.

In our cohort, IVOS was consistently associated with more favorable clinical outcomes. Reduced LOS with IVOS has been found in numerous previous studies [8, 9, 23–25, 34], even though the reasons behind this association have not been fully clarified. Tamma et al [23] argued that, in their US-based cohort, challenges associated with organizing outpatient parenteral antibiotic therapy may have delayed discharge, thereby prolonging LOS in patients with continued intravenous therapy. Structures for outpatient parenteral antibiotic therapy were lacking in Germany at the time of study conduct, consequently preventing discharge on intravenous therapy [35]. Yet we could not determine whether it was switch or discharge that drove the association between IVOS and LOS, that is, whether patients were discharged because they had been switched or whether they were switched because of a planned discharge. However, the fact that the majority of switches occurred on or immediately before discharge suggests that reverse causation is likely, with discharge planning driving IVOS rather than IVOS enabling earlier discharge. Furthermore, only a minority of patients were switched by day 3, suggesting that IVOS decisions were frequently delayed beyond clinical stabilization. These findings indicate that IVOS appears to be used as an organizational step preceding discharge rather than as an early therapeutic optimization strategy and that, consequently, benefits of IVOS beyond facilitating discharge may not be realized.

In contrast, our finding of lower mortality rates in patients undergoing IVOS differs from findings of most previous studies, which did not detect an association between IVOS and mortality rate or treatment failure [9, 23, 25, 34]. Only 1 systematic review reported oral therapy to be associated with reduced mortality rate, attributing this effect to decreased harm of intravenous treatment, such as catheter-associated complications [6]. This alone is unlikely to explain the observed mortality rate difference in our cohort, especially in light of the magnitude of the observed effect. A more plausible explanation is confounding by indication, as patients in the IVOS group had a better clinical status than those maintained on intravenous therapy, resulting in a lower baseline risk of death. Although application of clinical criteria for safe IVOS and multivariable analysis may have lessened confounding [23], IVOS remained independently associated with reduced mortality rate, suggesting that the variables included in our multivariable model may not fully capture determinants of prognosis and clinical decision making. Therefore, these findings should not be interpreted as evidence of a causal effect of IVOS on mortality rate but rather as an indication of the treatment decisions made for patients with a more favorable prognosis.

Taken together, our study highlights the need to further promote IVOS. Suitable and effective AMS measures for this purpose are described in previous literature, including the implementation of local guidelines, ID case review, and education of physicians [7, 36, 37]. As the variation in IVOS rates between centers observed in our study suggests that IVOS practices are influenced not only by patient characteristics but also by local prescribing practices, such measures should be tailored to meet local needs. In our study, the variation in IVOS rates did not reflect the AMS measures implemented in the participating centers. Yet the collected information might not be detailed enough to determine the effect of these measures on patient treatment, and our study nonetheless highlights some areas with particular potential for AMS intervention.

One of these areas would be addressing persisting concerns about the safety of IVOS in more severely ill patients, including those with a higher burden of comorbidity. Furthermore, as our findings suggest that IVOS is frequently implemented as a discharge-related intervention, AMS programs should place particular emphasis on IVOS in eligible patients even if their discharge is not yet planned. Earlier implementation of IVOS could maximize the potential benefits associated with oral antibiotic therapy, including a reduction in catheter-associated infections, decreased staff workload, and reduced drug costs, which are also advantageous for the treating hospital. In addition, patients may benefit from confirming oral tolerability of antibiotic therapy before discharge to the home environment. A road map for facilitating IVOS may include choosing an empiric antibiotic with an oral equivalent when feasible, providing guidelines to facilitate choice of oral antibiotics, reevaluating the route of administration early, and integrating IVOS into existing antibiotic de-escalation practices.

This study had some limitations. First, the analysis focused on evaluation of IVOS among stable patients with an evident option for oral therapy. While our exclusion criteria followed standard recommendations for markers of clinical stability required before making the switch in severe infections such as BSI [3, 33], it is likely that IVOS would have been safe in some of the excluded patients. Second, recent evidence published since the end of data collection suggests that oral antibiotics may be an adequate choice for some conditions not considered in the present analysis, including *S aureus* BSI in select patients [12, 38], infective endocarditis [39], and bone and joint infection [40, 41]. However, these infections were excluded to analyze the adherence to IVOS recommendations available at the time of the study. Third, our dataset did not capture several relevant factors that influence clinical decisions regarding IVOS, including the ability to swallow, gastrointestinal absorption, anticipated surgical interventions, detailed information on source control, and potential interactions with drugs other than antibiotics. As a consequence, eligibility for IVOS may have been overestimated in our analysis. At the same time, AST results were collected only for selected antibiotics. Limited information on narrow-spectrum β-lactam antibiotics with potential use in gram-negative infection might have led to underestimation of IVOS options.

As a fourth limitation, IVOS evaluation on day 7 may not fully reflect current practice as shorter treatment durations are increasingly being discussed for gram-negative BSI. Still, longer treatment courses were present in our cohort, and the chosen time point allowed assessment of factors influencing IVOS after clinical stabilization. Fifth, relevant infection-related outcomes, including microbiological cure, recurrence of BSI, and treatment failure, were not assessed in our analysis. Finally, the study period extended into the coronavirus disease 2019 (COVID-19) pandemic, potentially influencing AMS practices. However, IVOS rates and LOS showed no relevant changes for 2020 compared with previous years, and BSI secondary to respiratory infection was uncommon in our cohort. We therefore believe that our findings were not substantially affected by COVID-19.

In conclusion, in a clinically stable cohort of patients with BSI, opportunities for switching to oral antibiotics were frequently missed, with significant differences between the characteristics of patients with and without IVOS. IVOS was associated with markers of a more favorable prognosis, likely reflecting clinician selection of lower-risk patients for the switch. While our study did not measure the concerns of physicians refraining from IVOS, it seems likely that low IVOS rates may be driven by limited familiarity with the safety and benefits of IVOS. Our findings should serve as an impetus to strengthen AMS efforts in our setting.

## Supplementary Material

ofag666_Supplementary_Data

## Supplementary Data

Supplementary materials are available at *Open Forum Infectious Diseases* online. Consisting of data provided by the authors to benefit the reader, the posted materials are not copyedited and are the sole responsibility of the authors, so questions or comments should be addressed to the corresponding author.
