# Supplementary material for: Prevalence, Determinants and Outcomes of Intravenous-to-Oral Switch of Antibiotic Therapy in Hospitalized Adults With Bloodstream Infection: Secondary Analysis of a Multicenter Cohort
Source: Open Forum Infect Dis. 2026 Oct 19;13(11):ofag666. doi: 10.1093/ofid/ofag666 (PMC13749178; doi:10.1093/ofid/ofag666)
Supplement: Supplementary file 1 [file ofag666_supplementary_data.docx]

**Supplementary Material for**

**Prevalence, determinants and outcomes of intravenous to oral switch of antibiotic therapy in hospitalized adults with bloodstream infection: secondary analysis of a multicenter cohort**

**Authors:**

Lea A. Nikolai, Beryl P. Gladstone, Sarah V. Walker, Jörg J. Vehreschild, Kristina Schmauder, Simone Eisenbeis, Alexander Mischnik, Evelyn Kramme, Christine Geffers, Siegbert Rieg, Trinad Chakraborty, Maria J.G.T. Vehreschild, Harald Seifert, Jan Rupp, Silke Peter, Winfried V. Kern, Evelina Tacconelli, Daniel Hornuss, Siri Göpel, and the BLOOMY.COM study group

**Table of contents**

[**List of abbreviations**	3](#_Toc230874044)

[**S Table 1: BLOOMY and BLOOMY-PREDICT cohort studies**	4](#_Toc230874045)

[**Supplementary Methods section**	5](#_Toc230874046)

[**S Table 2: STROBE Statement**	7](#_Toc230874047)

[**S Table 3: Full list of comorbidities based on the Charlson Comorbidity Index among included patients**	9](#_Toc230874048)

[**S Table 4: Patient characteristics among study centers (among patients with an option for switch, n=742)**	10](#_Toc230874049)

[**S Table 5: Center-wise IVOS-rates by focus of infection (among patients with an option for switch, n=742)**	12](#_Toc230874050)

[**S Table 6: Center-wise IVOS-rates by pathogen (among patients with an option for switch, n=742)**	12](#_Toc230874051)

[**S Table 7: Multivariable logistic regression analysis: Factors associated with IVOS among all patients eligible for switch (n=742)**	13](#_Toc230874052)

[**S Table 8: Patient characteristics in patients with early vs. late switch (n=240)**	16](#_Toc230874053)

[**S Table 9: Most used antibiotics before switch among those with IVOS, full list (n=240)**	18](#_Toc230874054)

[**S Table 10: Most used antibiotic classes before switch among those with IVOS (n=240)**	18](#_Toc230874055)

[**S Table 11: Most used antibiotics after switch among those with IVOS, full list (n=240)**	18](#_Toc230874056)

[**S Table 12: Most used antibiotic classes after switch among those with IVOS (n=240)**	19](#_Toc230874057)

[**S Table 13: Most used empiric antibiotics among those with an option for IVOS (n=742)**	19](#_Toc230874058)

[**S Table 14: Most used day 7 antibiotics among those with an option for IVOS (n=742)**	19](#_Toc230874059)

[**S Figure 1: Most frequently used antibiotics and change in their use in patients switched to oral therapy by day 7 (n=240)**	20](#_Toc230874060)

[**S Figure 2: Most frequently used antibiotics and change in their use in patients not switched to oral therapy by day 7 (n=502)**	20](#_Toc230874061)

[**S Table 15: Final multivariable mixed effect logistic regression model of factors associated with 28-day mortality (n=644^a^)**	21](#_Toc230874062)

# **List of abbreviations**

AIDS	Acquired immune deficiency syndrome

BC	Blood culture

BSI	Bloodstream infection

EUCAST	European Committee on Antimicrobial Susceptibility Testing

GFR	Glomerular filtration rate

HIV	Human immunodeficiency virus

ICU	Intensive care unit

IV	Intravenous

IVOS	Intravenous to oral switch

MDR	Multidrug resistance

OR	Odds ratio

STROBE	Strengthening the reporting of observational studies in epidemiology

# **S Table 1: BLOOMY and BLOOMY-PREDICT cohort studies**

| **Databases** | **BLOOMY cohort** | **BLOOMY-PREDICT cohort** |
| --- | --- | --- |
| **General** |  |  |
| Exclusion criteria | Admission to an ophthalmology or psychiatry department | |
| Study centers | 6 tertiary care hospitals:  Berlin, Freiburg, Giessen, Cologne, Lübeck, and Tübingen | 5 tertiary care hospitals  Berlin, Freiburg, Cologne, Lübeck, and Tübingen |
| Study period | Feb 1, 2017-Jan 31, 2019 | Oct 9, 2019-Dec 31, 2020 |
| Patients in the database | 2627 | 1197 |
| Death within 3 days | 195 | 148 |
| **Differing definitions used - due to lack of similar variables:** |  |  |
| Co-infection with non-ESKAPE pathogen | Data not collected, therefore rate of polymicrobial infection might be underestimated | Data collected |
| Immunosuppressive therapy | Immunosuppressive therapy on day 1 - 3, cortisone therapy at any time during the stay | Cortisone therapy at any time during the stay for immunosuppressive use |
|  | Chemotherapy during hospitalization, neutropenia (< 500/µL) on day 3 and/or day 7 (If neutrophil count is not available: Leukopenia (< 1000/ µL) on day 3 and day 7), AIDS diagnosis | |
| Antibiotic allergies | Data not collected | Data collected |
| Antibiogram | Gram-positive: ampicillin, teicoplanin, vancomycin, linezolid, imipenem, meropenem, ciprofloxacin, gentamicin, cotrimoxazole, penicillin, oxacillin | Gram-positive: ampicillin, teicoplanin, vancomycin, linezolid, penicillin, oxacillin |
|  | Gram-negative: piperacillin/tazobactam, cefotaxime, ceftazidime, ertapenem, imipenem, meropenem, ciprofloxacin, gentamicin, cotrimoxazole | |
| **Uncollected data** | Presence of source control, microbiological findings before day 0, positive BCs between day 0 and day 3, results of microbiological samples other than BCs | |

# **Supplementary Methods section**

Missing data management

Since our data analyses involved assessment of clinical decision making based on available data, imputing methods for missing data values were not applied in our dataset.

Antibiotic data management

As the BLOOMY database contains data on all prescribed antibiotics independent of indication, all antibiotics administered for reasons other than BSI were removed from the database, such as for prophylactic antibiotic administration (antibiotics in which “end of prophylactic antibiotic use” was stated as the reason for stopping this specific therapy; prophylactic cotrimoxazole, referring to 480 – 960 mg on alternating days), oral vancomycin and fidaxomicin (for *C. difficile* infection), antibiotic combinations used for selective decontamination of the digestive tract (combination therapy of tobramycin, colistin and amphotericin B). Single-shot antibiotics were also removed (antibiotics labelled as “single shot” in the PREDICT database; dosing category “single shot” in BLOOMY; antibiotics that were given only once on a given day and not within the two days before or after). Other removed antibiotics were rifaximin, oral and inhalative colistin, oral fosfomycin, and metronidazole.

The last day of antibiotic treatment was defined as the last day on which the patient received antibiotics with no antibiotic treatment during the following two days or as the last day of hospitalization if antibiotics were continued until discharge and no antibiotic therapy was recommended in the discharge letter. Antibiotics prescribed upon hospital discharge were considered for analysis if a recommendation on the further duration of therapy was given in discharge notes.

When there was an antibiotic overlap (i.e. the day on which a single or multiple antibiotics were stopped and a new antibiotic was introduced), we considered the antibiotic therapy that was continued after as the main antibiotic therapy during the concerned day. Among patients receiving multiple antibiotics on day 3 or 7, it was checked if both IV and oral antibiotics were given on that day only (=overlap of 1 day). In this case, we assumed that the route of administration was readjusted on the respective day. The “newer” antibiotic (started on day 3 or 7) was the antibiotic that evaluation of switch was based on. If the overlap was more than one day, the therapy was not considered switched.

Only antibiotics reported as “S” in sensitivity testing based on European Committee on Antimicrobial Susceptibility Testing breakpoints were considered to cover the detected pathogen, as the new EUCAST definition of I (increased dosage) was not introduced until the end of the study. When determining if an option for IVOS was indicated by the antibiogram, we additionally checked if allergies prevented switch to the antibiotics tested as “sensitive”.

Availability of oral alternatives

Antibiogram data for antibiotics which can be administered orally was available for the following substances: For patients with Gram-negative infection, it was checked it ciprofloxacin and/or cotrimoxazole were reported as sensitive. In patients infected with *P. aeruginosa*, only ciprofloxacin was considered. In patients with Gram-positive infection, it was checked if ampicillin and/or linezolid were reported as sensitive. Testing for other orally available antibiotics was not available in our database.

IV antibiotics considered to have an oral equivalent with sufficient bioavailability were levofloxacin, moxifloxacin, amoxicillin/clavulanic acid, clindamycin, cefalexin, cefpodoxime, clarithromycin, cotrimoxazole, linezolid, amoxicillin and penicillin.

Study definitions

Monomicrobial BSI was considered to be present when the database indicated no co- or superinfection either with an ESKAPE or non-ESKAPE pathogen concomitantly or in subsequent blood cultures until day 14.

Immunosuppression was defined as neutropenia (neutrophils < 500/µL) on at least 2 days out of day 0, day 3 and day 7 (if the neutrophil count was not available, leukocyte count < 1000/ µL was used instead), administration of cortisone therapy outside the ICU between day 4 and 14 (for BLOOMY, any cortisone treatment was recorded while the PREDICT database contains cortisone use for immunosuppressive use only), chemotherapy between day 0 and day 7, cotrimoxazole in prophylactic dosages (800mg /160 on alternate days, 480 mg on alternate days or daily in patients with a GFR > 30 ml/min) or diagnosis of HIV in AIDS stadium. In the BLOOMY cohort, information on administration of immunosuppressive drugs other than cortisone was additionally used.

Community-acquired infection (CAI) was present when the positive BC was drawn up to the day after hospital admission and healthcare-associated infection (HCAI) was defined as patients who had received inpatient treatment in a hospital, nursing home, rehabilitation facility or outpatient clinic treatment for chemotherapy or dialysis within the 3 months prior to admission. Other infections were considered hospital-acquired (HAI).

Multidrug-resistant (MDR) infection included Gram-negative pathogens with resistance to at least 3 out 4 antibiotic classes (piperacillin, cefotaxime or ceftazidime, ciprofloxacin, imipenem or meropenem), methicillin-resistant *S. aureus* (MRSA), and vancomycin-resistant *Enterococcus* (VRE).

# **S Table 2: STROBE Statement**

Checklist of items that should be included in reports of ***cohort studies.***

|  | Item No | Recommendation | Page No |
| --- | --- | --- | --- |
| **Title and abstract** | 1 | (*a*) Indicate the study’s design with a commonly used term in the title or the abstract | 1 |
|  |  | (*b*) Provide in the abstract an informative and balanced summary of what was done and what was found | 4 |
| Introduction | | |  |
| Background/ rationale | 2 | Explain the scientific background and rationale for the investigation being reported | 5 |
| Objectives | 3 | State specific objectives, including any prespecified hypotheses | 5 |
| Methods | | |  |
| Study design | 4 | Present key elements of study design early in the paper | 5 |
| Setting | 5 | Describe the setting, locations, and relevant dates, including periods of recruitment, exposure, follow-up, and data collection | 5-6 |
| Participants | 6 | (*a*) Give the eligibility criteria, and the sources and methods of selection of participants. Describe methods of follow-up | 5-8 |
|  |  | (*b*) For matched studies, give matching criteria and number of exposed and unexposed | n/a |
| Variables | 7 | Clearly define all outcomes, exposures, predictors, potential confounders, and effect modifiers. Give diagnostic criteria, if applicable | 6-8 |
| Data sources/ measurement | 8* | For each variable of interest, give sources of data and details of methods of assessment (measurement). Describe comparability of assessment methods if there is more than one group | n/a |
| Bias | 9 | Describe any efforts to address potential sources of bias | 6-8 |
| Study size | 10 | Explain how the study size was arrived at | n/a |
| Quantitative variables | 11 | Explain how quantitative variables were handled in the analyses. If applicable, describe which groupings were chosen and why | 8 |
| Statistical methods | 12 | (*a*) Describe all statistical methods, including those used to control for confounding | 8 |
|  |  | (*b*) Describe any methods used to examine subgroups and interactions | n/a |
|  |  | (*c*) Explain how missing data were addressed | Suppl. 5 |
|  |  | (*d*) If applicable, explain how loss to follow-up was addressed | n/a |
|  |  | (*e*) Describe any sensitivity analyses | n/a |
| Results | | |  |
| Participants | 13 | (a) Report numbers of individuals at each stage of study—eg numbers potentially eligible, examined for eligibility, confirmed eligible, included in the study, completing follow-up, and analysed | Figure 1 |
|  |  | (b) Give reasons for non-participation at each stage | Figure 1 |
|  |  | (c) Consider use of a flow diagram | Figure 1 |
| Descriptive data | 14 | (a) Give characteristics of study participants (eg demographic, clinical, social) and information on exposures and potential confounders | Table 1 |
|  |  | (b) Indicate number of participants with missing data for each variable of interest | Table 1 |
|  |  | (c) Summarise follow-up time (eg, average and total amount) | n/a |
| Outcome data | 15 | Report numbers of outcome events or summary measures over time | 9-16 |
| Main results | 16 | (*a*) Give unadjusted estimates and, if applicable, confounder-adjusted estimates and their precision (eg, 95% confidence interval). Make clear which confounders were adjusted for and why they were included | Table 2, Table 3, Suppl Table 7 |
|  |  | (*b*) Report category boundaries when continuous variables were categorized | 8-16 |
|  |  | (*c*) If relevant, consider translating estimates of relative risk into absolute risk for a meaningful time period | n/a |
| Other analyses | 17 | Report other analyses done—eg analyses of subgroups and interactions, and sensitivity analyses | n/a |
| Discussion | | |  |
| Key results | 18 | Summarise key results with reference to study objectives | 16 |
| Limitations | 19 | Discuss limitations of the study, taking into account sources of potential bias or imprecision. Discuss both direction and magnitude of any potential bias | 19-20 |
| Interpretation | 20 | Give a cautious overall interpretation of results considering objectives, limitations, multiplicity of analyses, results from similar studies, and other relevant evidence | 20 |
| Generalisability | 21 | Discuss the generalisability (external validity) of the study results | 16-20 |
| Other information | | |  |
| Funding | 22 | Give the source of funding and the role of the funders for the present study and, if applicable, for the original study on which the present article is based | 21 |

# **S Table 3: Full list of comorbidities based on the Charlson Comorbidity Index among included patients**

|  | **Included in secondary analysis**  **(n=2770)^a^** | | **Final study cohort of those eligible and evaluable for IVOS**  **(n=742)^a^** | |
| --- | --- | --- | --- | --- |
| **Variable** | **n** | **%** | **n** | **%** |
| Prior myocardial infarction | 243 | 8.9 | 62 | 8.5 |
| Congestive heart failure | 656 | 24.6 | 142 | 19.7 |
| Peripheral vascular disease | 274 | 10.2 | 58 | 8.0 |
| Cerebrovascular disease | 265 | 9.7 | 79 | 10.7 |
| Dementia | 114 | 4.2 | 49 | 6.7 |
| Chronic pulmonary disease | 415 | 15.3 | 100 | 13.6 |
| Connective tissue disease | 125 | 4.6 | 31 | 4.2 |
| Peptic ulcer disease | 158 | 5.8 | 40 | 5.5 |
| Mild liver disease | 206 | 7.6 | 48 | 6.6 |
| Moderate-to-severe liver disease | 178 | 6.5 | 47 | 6.4 |
| Uncomplicated diabetes | 494 | 18.0 | 142 | 19.3 |
| Diabetes with chronic complications | 258 | 9.4 | 64 | 8.8 |
| Cerebrovascular event with Hemiplegia | 85 | 3.1 | 16 | 2.2 |
| Moderate-to-severe kidney disease | 796 | 29.2 | 191 | 26.2 |
| Localized solid tumor | 1203 | 44.3 | 285 | 39.4 |
| Metastatic solid tumor | 418 | 15.9 | 125 | 17.8 |
| Leukemia | 165 | 8.2 | 8 | 1.5 |
| Lymphoma | 153 | 7.6 | 11 | 2.1 |

a. Denominators varied due to missing values. Total numbers for respective variables were: prior myocardial infarction (n=2730, n=733), congestive heart failure (n=2662, n=719), peripheral vascular disease (n=2682, n=723), cerebrovascular disease (n=2736, n=735), dementia (n=2700, n=732), chronic pulmonary disease (n=2714, n=733), connective tissue disease (n=2744, n=736), peptic ulcer disease (n=2709, n=727), mild liver disease (n=2705, n=730), moderate-to-severe liver disease (n=2721, n=731), uncomplicated diabetes (n=2746, n=734), diabetes with chronic complications (n=2732, n=731), cerebrovascular event with hemiplegia (n=2742, n=735), moderate-to-severe chronic kidney disease (n=2730, n=730), localized solid tumor (n=2718, n=723), metastatic solid tumor (n=2623, n=703), leukemia (n=2015, n=524), lymphoma (n=2015, n=523)

Abbreviations: IVOS – intravenous to oral switch

# **S Table 4: Patient characteristics among study centers (among patients with an option for switch, n=742)**

|  | Center 1 | | Center 2 | | Center 3 | | Center 4 | | Center 5 | | Center 6 | | Total | |
| --- | --- | --- | --- | --- | --- | --- | --- | --- | --- | --- | --- | --- | --- | --- |
| **Variable** | **n** | **%** | **n** | **%** | **n** | **%** | **n** | **%** | **n** | **%** | **n** | **%** | **n** | **%** |
| Female sex | 37 | 44.0 | 100 | 40.7 | 3 | 27.3 | 16 | 38.1 | 82 | 42.7 | 72 | 44.4 | 310 | 42.1 |
| Age (years) |  |  |  |  |  |  |  |  |  |  |  |  |  |  |
| ≤ 40 | 3 | 3.4 | 11 | 4.5 | - | - | - | - | 6 | 3.1 | 9 | 5.6 | 29 | 3.9 |
| 41 - 60 | 11 | 12.5 | 60 | 24.3 | 4 | 36.4 | 15 | 35.7 | 25 | 13.0 | 30 | 18.5 | 145 | 19.5 |
| 61 - 80 | 55 | 62.5 | 132 | 53.4 | 7 | 63.6 | 20 | 47.6 | 101 | 52.6 | 86 | 53.1 | 401 | 54.0 |
| > 80 | 19 | 21.6 | 44 | 17.8 | - | - | 7 | 16.7 | 60 | 31.3 | 37 | 22.8 | 167 | 22.5 |
| Charlson Comorbidity Index ≥ 3 | 41 | 47.1 | 94 | 38.4 | 3 | 33.3 | 24 | 58.5 | 45 | 23.9 | 50 | 30.9 | 257 | 35.1 |
| Malignancy | 40 | 45.5 | 112 | 45.3 | 5 | 50.0 | 31 | 73.8 | 41 | 21.5 | 56 | 34.6 | 285 | 38.5 |
| Nursing home residence | 3 | 3.4 | 2 | 0.8 | - | - | - | - | 26 | 13.5 | 5 | 3.1 | 36 | 4.9 |
| ICU admission in the last 3 months | 7 | 8.6 | 25 | 10.7 | - | - | 4 | 9.5 | 11 | 6.1 | 10 | 7.2 | 57 | 8.3 |
| In ICU on day 0 | 18 | 20.5 | 38 | 15.4 | - | - | 4 | 9.5 | 14 | 7.3 | 17 | 10.5 | 91 | 12.3 |
| Pitt Bacteremia Score day 0 |  |  |  |  |  |  |  |  |  |  |  |  |  |  |
| < 2 | 35 | 71.4 | 113 | 73.4 | 10 | 90.9 | 17 | 77.3 | 107 | 72.8 | 70 | 71.4 | 352 | 73.2 |
| ≥ 2 | 14 | 28.6 | 41 | 26.6 | 1 | 9.1 | 5 | 22.7 | 40 | 27.2 | 28 | 28.6 | 129 | 26.8 |
| Pitt Bacteremia Score day 3 |  |  |  |  |  |  |  |  |  |  |  |  |  |  |
| < 2 | 73 | 90.1 | 213 | 89.9 | 11 | 100.0 | 39 | 95.1 | 155 | 97.5 | 133 | 84.2 | 624 | 90.8 |
| ≥ 2 | 8 | 9.9 | 24 | 10.1 | - | - | 2 | 4.9 | 4 | 2.5 | 25 | 15.8 | 63 | 9.2 |
| Site of acquisition |  |  |  |  |  |  |  |  |  |  |  |  |  |  |
| Community-acquired | 24 | 27.3 | 77 | 31.2 | 2 | 18.2 | 7 | 16.7 | 62 | 32.3 | 55 | 34.0 | 227 | 30.6 |
| Healthcare-associated | 46 | 52.3 | 85 | 34.4 | 5 | 45.5 | 18 | 42.9 | 90 | 46.9 | 82 | 50.6 | 326 | 43.9 |
| Hospital-acquired | 18 | 20.5 | 85 | 34.4 | 4 | 36.4 | 17 | 40.5 | 40 | 20.8 | 25 | 15.4 | 189 | 25.5 |
| History of MDR infection in the last 3 months | 4 | 4.6 | 17 | 6.9 | - | - | 2 | 4.8 | 5 | 2.6 | 9 | 5.6 | 37 | 5.0 |
| Focus of infection |  |  |  |  |  |  |  |  |  |  |  |  |  |  |
| Abdominal | 18 | 20.5 | 76 | 30.8 | 3 | 27.3 | 17 | 40.5 | 34 | 17.7 | 35 | 21.6 | 183 | 24.7 |
| Urogenital | 45 | 51.1 | 114 | 46.2 | 7 | 63.6 | 13 | 31.0 | 111 | 57.8 | 85 | 52.5 | 375 | 50.5 |
| Respiratory | 10 | 11.4 | 8 | 3.2 | - | - | - | - | 25 | 13.0 | 3 | 1.9 | 46 | 6.2 |
| Cardiovascular | - | - | 8 | 3.2 | - | - | 5 | 11.9 | 1 | 0.5 | 2 | 1.2 | 16 | 2.2 |
| Skin and soft tissue | 1 | 1.1 | 4 | 1.6 | 1 | 9.1 | - | - | 6 | 3.1 | 4 | 2.5 | 15 | 2.0 |
| Multiple foci | 3 | 3.4 | 6 | 2.4 | - | - | 1 | 2.4 | 7 | 3.7 | 4 | 2.5 | 21 | 2.8 |
| Unknown focus | 11 | 12.5 | 31 | 12.6 | - | - | 6 | 14.3 | 8 | 4.2 | 30 | 18.5 | 86 | 11.6 |
| Foreign body infection | 3 | 3.4 | 21 | 8.5 | - | - | 9 | 21.4 | 7 | 3.7 | 3 | 1.9 | 43 | 5.8 |
| BSI pathogen |  |  |  |  |  |  |  |  |  |  |  |  |  |  |
| *Enterobacter (Klebsiella) aerogenes* | 3 | 3.4 | 4 | 1.6 | - | - | - | - | - | - | 1 | 0.6 | 8 | 1.1 |
| *Enterobacter cloacae* complex | - | - | 13 | 5.3 | 1 | 9.1 | 4 | 9.5 | 12 | 6.3 | 10 | 6.2 | 40 | 5.4 |
| *Enterococcus faecalis* | 13 | 14.8 | 21 | 8.5 | - | - | 3 | 7.1 | 19 | 9.9 | 8 | 4.9 | 64 | 8.6 |
| *Enterococcus faecium* | 4 | 4.5 | 21 | 8.5 | - | - | 6 | 14.3 | 10 | 5.2 | 8 | 4.9 | 49 | 6.6 |
| *Escherichia coli* | 45 | 51.1 | 137 | 55.5 | 8 | 72.7 | 20 | 47.6 | 123 | 64.1 | 112 | 69.1 | 445 | 60.0 |
| *Klebsiella oxytoca* | 2 | 2.3 | 13 | 5.3 | 1 | 9.1 | 3 | 7.1 | 3 | 1.6 | 3 | 1.9 | 25 | 3.4 |
| *Klebsiella pneumoniae* | 20 | 22.7 | 30 | 12.1 | - | - | 3 | 7.1 | 18 | 9.4 | 15 | 9.3 | 86 | 11.6 |
| *Pseudomonas aeruginosa* | 1 | 1.1 | 8 | 3.2 | 1 | 9.1 | 2 | 4.8 | 6 | 3.1 | 4 | 2.5 | 22 | 3.0 |
| Other | - | - | - | - | - | - | 1 | 2.4 | 1 | 0.5 | 1 | 0.6 | 3 | 0.4 |
| MDR pathogen | 3 | 3.4 | 14 | 5.7 | - | - | 2 | 4.8 | 12 | 6.3 | 9 | 5.6 | 40 | 5.4 |
| Adverse Events (after day 7) |  |  |  |  |  |  |  |  |  |  |  |  |  |  |
| Septic Shock | 1 | 1.2 | 3 | 1.2 | - | - | - | - | - | - | 1 | 0.6 | 5 | 0.7 |
| Acute kidney injury | 3 | 4.0 | 4 | 1.7 | - | - | - | - | 1 | 0.6 | 1 | 0.6 | 9 | 1.3 |
| Mortality |  |  |  |  |  |  |  |  |  |  |  |  |  |  |
| In-Hospital | 10 | 11.4 | 18 | 7.3 | - | - | 1 | 2.4 | 12 | 6.3 | 4 | 2.5 | 45 | 6.1 |
| 28-day | 11 | 12.5 | 24 | 9.7 | - | - | 1 | 2.4 | 14 | 7.3 | 5 | 3.1 | 55 | 7.4 |
| 6-month | 14 | 18.4 | 50 | 24.2 | 1 | 10.0 | 7 | 21.9 | 30 | 27.8 | 24 | 17.9 | 126 | 22.2 |
| Median Length of Stay (after positive blood culture) [p25;p75] | 11 [9;17] | | 11 [9;17] | | 8 [8;12] | | 9 [12;18] | | 10 [8;14] | | 9 [7;13] | | 10 [8;15] | |

Abbreviations: BSI – bloodstream infection, ICU – intensive care unit, MDR – multidrug resistant

# **S Table 5: Center-wise IVOS-rates by focus of infection (among patients with an option for switch, n=742)**

|  | **Center 1** | **Center 2** | **Center 3** | **Center 4** | **Center 5** | **Center 6** | **Total** |
| --- | --- | --- | --- | --- | --- | --- | --- |
| **Total number of patients included in the respective center** | 88 | 247 | 11 | 42 | 192 | 162 | 743 |
|  | **n (%)** | **n (%)** | **n (%)** | **n (%)** | **n (%)** | **n (%)** | **n (%)** |
| **Abdominal** | 1 (5.6) | 11 (14.5) | 2 (66.7) | 5 (29.4) | 11 (32.4) | 15 (42.9) | 45 (24.6) |
| **Urogenital** | 15 (33.3) | 32 (28.1) | 2 (28.6) | 4 (30.8) | 46 (41.4) | 55 (64.7) | 154 (41.1) |
| **Respiratory** | 0 (0.0) | 0 (0.0) | - | - | 4 (16.0) | 1 (33.3) | 5 (10.9) |
| **Cardiovascular** | - | 0 (0.0) | - | 2 (40.0) | 0 (0.0) | 1 (50.0) | 3 (18.8) |
| **Skin and Soft Tissue** | 0 (0.0) | 0 (0.0) | 0 (0.0) | - | 2 (33.3) | 2 (66.7) | 4 (26.7) |
| **Multiple foci** | 0 (0.0) | 1 (16.7) | - | 0 (0.0) | 1 (14.3) | 1 (25.0) | 3 (14.3) |
| **Unknown focus** | 0 (0.0) | 7 (22.6) | - | 1 (16.7) | 1 (12.5) | 17 (56.7) | 26 (30.2) |
| **Total** | 16 (18.2) | 51 (20.6) | 4 (36.4) | 12 (28.6) | 65 (33.9) | 92 (56.8) | 240 (32.3) |

# **S Table 6: Center-wise IVOS-rates by pathogen (among patients with an option for switch, n=742)**

|  | **Center 1** | **Center 2** | **Center 3** | **Center 4** | **Center 5** | **Center 6** | **Total** |
| --- | --- | --- | --- | --- | --- | --- | --- |
| **Total number of patients included in the respective center** | 88 | 247 | 11 | 42 | 192 | 162 | 743 |
|  | **n (%)** | **n (%)** | **n (%)** | **n (%)** | **n (%)** | **n (%)** | **n (%)** |
| ***Enterobacter (Klebsiella) aerogenes*** | 0 (0.0) | 0 (0.0) | - | - | - | 1 (100.0) | 1 (12.5) |
| ***Enterobacter cloacae* complex** | - | 3 (23.1) | 0 (0.0) | 3 (75.0) | 4 (33.3) | 4 (40.0) | 14 (35.0) |
| ***Enterococcus faecalis*** | 5 (38.5) | 4 (19.0) | - | 1 (33.3) | 7 (36.8) | 4 (50.0) | 21 (32.8) |
| ***Enterococcus faecium*** | 0 (0.0) | 0 (0.0) | - | 2 (33.3) | 1 (10.0) | 1 (12.5) | 4 (8.2) |
| ***Escherichia coli*** | 9 (20.0) | 37 (27.0) | 4 (50.0) | 4 (20.0) | 44 (35.8) | 66 (58.9) | 164 (36.9) |
| ***Klebsiella oxytoca*** | 0 (0.0) | 1 (7.7) | 0 (0.0) | 1 (33.3) | 2 (66.7) | 2 (66.7) | 6 (24.0) |
| ***Klebsiella pneumoniae*** | 2 (10.0) | 6 (20.0) | - | - | 6 (33.3) | 11 (73.3) | 25 (29.1) |
| ***Pseudomonas aeruginosa*** | 0 (0.0) | 0 (0.0) | 0 (0.0) | 1 (50.0) | 1 (16.7) | 3 (75.0) | 5 (22.7) |
| **Other** | - | - | - | 0 (0.0) | 0 (0.0) | 0 (0.0) | 0 (0.0) |
| **Total** | 16 (18.2) | 51 (20.6) | 4 (36.4) | 12 (28.6) | 65 (33.9) | 92 (56.8) | 240 (32.3) |

# **S Table 7: Multivariable logistic regression analysis: Factors associated with IVOS among all patients eligible for switch (n=742)**

|  | **Total** | **Switch** | **Non-Switch** | **Univariable Analysis** | | **Multivariable Full Model^a^** | |
| --- | --- | --- | --- | --- | --- | --- | --- |
|  | n | n (%) | n (%) | unadjusted odds ratio (OR) with 95% CI | p-value | adjusted odds ratio (OR) with 95% CI | p-value |
| **Variable** |  |  |  |  |  |  |  |
| Age (years) |  |  |  |  |  |  |  |
| ≤ 40 | 29 | 15 (51.7) | 14 (48.3) | 1 |  | 1 |  |
| 41 - 60 | 145 | 42 (29.0) | 103 (71.0) | 0.38 (0.17-0.86) | 0.020 | 0.38 (0.13-1.06) | 0.065 |
| 61 - 80 | 401 | 136 (33.9) | 265 (66.1) | 0.48 (0.22-1.02) | 0.057 | 0.64 (0.24-1.71) | 0.371 |
| > 80 | 167 | 47 (28.1) | 120 (71.9) | 0.37 (0.16-0.82) | 0.014 | 0.35 (0.12-1.00) | 0.050 |
| Moderate-to-severe kidney disease |  |  |  |  |  |  |  |
| No | 539 | 189 (35.1) | 350 (64.9) | 1 |  | 1 |  |
| Yes | 191 | 50 (26.2) | 141 (73.8) | 0.66 (0.45-0.95) | 0.025 | 0.66 (0.41-1.08) | 0.099 |
| Moderate-to-severe-liver disease |  |  |  |  |  |  |  |
| No | 684 | 227 (33.2) | 457 (66.8) | 1 |  | 1 |  |
| Yes | 47 | 10 (21.3) | 37 (78.7) | 0.54 (0.27-1.11) | 0.096 | 0.79 (0.31-2.05) | 0.633 |
| Congestive heart failure |  |  |  |  |  |  |  |
| No | 577 | 203 (35.2) | 374 (64.8) | 1 |  | 1 |  |
| Yes | 142 | 33 (23.2) | 109 (76.7) | 0.56 (0.36-0.85) | 0.007 | 0.62 (0.34-1.14) | 0.125 |
| Diabetes |  |  |  |  |  |  |  |
| No | 536 | 190 (35.4) | 346 (64.6) | 1 |  | 1 |  |
| Yes | 206 | 50 (24.3) | 156 (75.7) | 0.58 (0.41-0.84) | 0.004 | 0.49 (0.31-0.79) | 0.003 |
| Solid tumor |  |  |  |  |  |  |  |
| No | 438 | 155 (35.4) | 283 (64.6) | 1 |  | 1 |  |
| Yes | 285 | 76 (26.7) | 209 (73.3) | 0.66 (0.48-0.92) | 0.014 | 0.85 (0.47-1.54) | 0.602 |
| Metastasized solid tumor |  |  |  |  |  |  |  |
| No | 578 | 199 (34.4) | 379 (65.6) | 1 |  | 1 |  |
| Yes | 125 | 28 (22.4) | 97 (77.6) | 0.55 (0.35-0.87) | 0.010 | 0.80 (0.36-1.76) | 0.572 |
| Inflammatory bowel disease |  |  |  |  |  |  |  |
| No | 670 | 217 (32.4) | 453 (67.6) | 1 |  | 1 |  |
| Yes | 25 | 4 (16.0) | 21 (84.0) | 0.40 (0.13-1.17) | 0.095 | 0.29 (0.07-1.12) | 0.073 |
| Charlson Comorbidity Index |  |  |  |  |  |  |  |
| < 3 | 475 | 175 (36.8) | 300 (63.2) | 1 |  | 1 |  |
| ≥ 3 | 257 | 61 (23.7) | 196 (76.3) | 0.53 (0.38-0.75) | <0.001 | 1.00 (0.49-2.06) | 0.997 |
| ICU admission in the last 3 months |  |  |  |  |  |  |  |
| No | 626 | 215 (34.3) | 411 (65.7) | 1 |  | 1 |  |
| Yes | 57 | 11 (19.3) | 46 (80.7) | 0.46 (0.23-0.90) | 0.024 | 0.49 (0.21-1.16) | 0.104 |
| In ICU on day 0 |  |  |  |  |  |  |  |
| No | 651 | 226 (34.7) | 425 (65.3) | 1 |  | 1 |  |
| Yes | 91 | 14 (15.4) | 77 (84.6) | 0.34 (0.19-0.62) | <0.001 | 0.63 (0.28-1.40) | 0.258 |
| Pitt Bacteremia Score day 3 |  |  |  |  |  |  |  |
| < 2 | 624 | 213 (34.1) | 411 (65.9) | 1 |  | 1 |  |
| ≥ 2 | 63 | 10 (15.9) | 53 (84.1) | 0.36 (0.18-0.73) | 0.004 | 0.44 (0.18-1.05) | 0.063 |
| Septic shock before day 7 |  |  |  |  |  |  |  |
| No | 688 | 230 (33.4) | 458 (66.6) | 1 |  | 1 |  |
| Yes | 43 | 7 (16.3) | 36 (83.7) | 0.39 (0.17-0.88) | 0.024 | 0.33 (0.10-1.13) | 0.078 |
| Site of acquisition |  |  |  |  |  |  |  |
| Community-acquired | 227 | 81 (35.7) | 146 (64.3) | 1 |  | 1 |  |
| Healthcare-associated | 326 | 118 (36.2) | 208 (63.8) | 1.02 (0.72-1.46) | 0.902 | 1.45 (0.87-2.41) | 0.156 |
| Hospital-acquired | 189 | 41 (21.7) | 148 (78.3) | 0.50 (0.32-0.78) | 0.002 | 0.93 (0.50-1.73) | 0.820 |
| Gram stain |  |  |  |  |  |  |  |
| Gram-positive | 113 | 25 (22.1) | 88 (77.9) | 1 |  | 1 |  |
| Gram-negative | 629 | 215 (34.2) | 414 (65.8) | 1.83 (1.14-2.94) | 0.013 | 1.16 (0.61-2.23) | 0.650 |
| MDR pathogen |  |  |  |  |  |  |  |
| No | 702 | 233 (33.2) | 469 (66.8) | 1 |  | 1 |  |
| Yes | 40 | 7 (17.5) | 33 (82.5) | 0.43 (0.19-0.98) | 0.045 | 0.44 (0.15-1.31) | 0.139 |
| Focus of infection |  |  |  |  |  |  |  |
| Unknown | 86 | 26 (30.2) | 60 (69.8) | 1 |  | 1 |  |
| Abdominal | 183 | 45 (24.6) | 138 (75.4) | 0.75 (0.43-1.33) | 0.328 | 0.74 (0.36-1.53) | 0.419 |
| Respiratory | 46 | 5 (10.9) | 41 (89.1) | 0.28 (0.10-0.79) | 0.016 | 0.21 (0.04-1.06) | 0.059 |
| Urogenital | 375 | 154 (41.1) | 221 (58.9) | 1.61 (0.97-2.66) | 0.065 | 1.41 (0.73-2.74) | 0.303 |
| Cardiovascular | 16 | 3 (18.8) | 13 (81.3) | 0.53 (0.14-2.03) | 0.356 | 1.29 (0.20-8.36) | 0.791 |
| Skin, soft tissue | 15 | 4 (26.7) | 11 (73.3) | 0.84 (0.24-2.88) | 0.781 | 0.43 (0.07-2.72) | 0.373 |
| Multiple | 21 | 3 (14.3) | 18 (85.7) | 0.38 (0.10-1.42) | 0.152 | 0.40 (0.09-1.81) | 0.233 |
| Foreign body infection |  |  |  |  |  |  |  |
| No | 699 | 232 (33.2) | 467 (66.8) | 1 |  | 1 |  |
| Yes | 43 | 8 (18.6) | 35 (81.4) | 0.46 (0.21-1.00) | 0.052 | 0.51 (0.17-1.55) | 0.237 |
| Study center |  |  |  |  |  |  |  |
| Center 1 | 88 | 16 (18.2) | 72 (81.8) | 1 |  | 1 |  |
| Center 2 | 247 | 51 (20.6) | 196 (79.4) | 1.17 (0.63-2.18) | 0.620 | 0.95 (0.43-2.08) | 0.895 |
| Center 3 | 11 | 4 (36.4) | 7 (63.6) | 2.57 (0.67-9.85) | 0.168 | 2.08 (0.32-13.67) | 0.444 |
| Center 4 | 42 | 12 (28.6) | 30 (71.4) | 1.80 (0.76-4.28) | 0.008 | 2.19 (0.76-6.33) | 0.149 |
| Center 5 | 192 | 65 (33.9) | 127 (66.1) | 2.30 (1.24-4.28) | 0.008 | 1.62 (0.73-3.60) | 0.239 |
| Center 6 | 162 | 92 (56.8) | 70 (43.2) | 5.91 (3.17-11.04) | <0.001 | 6.21 (2.79-13.78) | <0.001 |
| Empirical therapy |  |  |  |  |  |  |  |
| Other | 572 | 170 (29.7) | 402 (70.3) | 1 |  | 1 |  |
| Empiric monotherapy with orally available antibiotic | 170 | 70 (41.2) | 100 (58.8) | 1.66 (1.16-2.36) | 0.005 | 1.18 (0.73-1.91) | 0.497 |

a. Data available for all the variables included in the full model for 626 of the 742 patients.

Abbreviations: ICU – intensive care unit, MDR – multidrug resistance

# **S Table 8: Patient characteristics in patients with early vs. late switch (n=240)**

|  | **early switch**  **(n =46, 19.2%)^a^** | | **late switch**  **(n = 194, 80.8%)^a^** | |  |
| --- | --- | --- | --- | --- | --- |
| **Variable** | **n** | **%** | **n** | **%** | **p-value** |
| Female sex | 20 | 18.7 | 87 | 81.3 | 0.822 |
| Age (years) |  |  |  |  |  |
| ≤ 40 | 5 | 33.3 | 10 | 66.7 | 0.374 |
| 41 - 60 | 10 | 23.8 | 32 | 76.2 |  |
| 61 - 80 | 23 | 16.9 | 113 | 83.1 |  |
| > 80 | 8 | 17.0 | 39 | 83.0 |  |
| Charlson Comorbidity Index ≥ 3 | 11 | 18.0 | 50 | 82.0 | 0.738 |
| Malignancy | 12 | 15.8 | 64 | 84.2 | 0.366 |
| Nursing home residence | 1 | 9.1 | 10 | 90.9 | 0.385 |
| ICU admission in the last 3 months | 4 | 36.4 | 7 | 63.6 | 0.147 |
| In ICU on day 0 | 4 | 28.6 | 10 | 71.4 | 0.357 |
| Pitt Bacteremia Score day 3 |  |  |  |  |  |
| < 2 | 46 | 21.6 | 167 | 78.4 | 0.099 |
| ≥ 2 | 0 | 0.0 | 10 | 100.0 |  |
| Site of acquisition |  |  |  |  |  |
| Community-acquired | 15 | 18.5 | 66 | 81.5 | 0.984 |
| Healthcare-associated | 23 | 19.5 | 95 | 80.5 |  |
| Hospital-acquired | 8 | 19.5 | 33 | 80.5 |  |
| History of MDR infection in the last 3 months | 1 | 10.0 | 9 | 90.0 | 0.452 |
| Focus of infection |  |  |  |  |  |
| Abdominal | 10 | 22.2 | 35 | 77.8 | 0.530 |
| Urogenital | 31 | 20.1 | 123 | 79.9 |  |
| Respiratory | 2 | 40.0 | 3 | 60.0 |  |
| Cardiovascular | 0 | 0.0 | 3 | 100.0 |  |
| Skin and soft tissue | 0 | 0.0 | 4 | 100.0 |  |
| Multiple foci | 0 | 0.0 | 3 | 100.0 |  |
| Unknown focus | 3 | 11.5 | 23 | 88.5 |  |
| Foreign body infection | 2 | 25.0 | 6 | 75.0 | 0.670 |
| BSI pathogen |  |  |  |  |  |
| *Enterobacter (Klebsiella) aerogenes* | 0 | 0.0 | 1 | 100.0 | 0.507 |
| *Enterobacter cloacae* complex | 2 | 14.3 | 12 | 85.7 |  |
| *Enterococcus faecalis* | 6 | 28.6 | 15 | 71.4 |  |
| *Enterococcus faecium* | 1 | 25.0 | 3 | 75.0 |  |
| *Escherichia coli* | 28 | 17.1 | 136 | 82.9 |  |
| *Klebsiella oxytoca* | 0 | 0.0 | 6 | 100.0 |  |
| *Klebsiella pneumoniae* | 8 | 32.0 | 17 | 68.0 |  |
| *Pseudomonas aeruginosa* | 1 | 20.0 | 4 | 80.0 |  |
| MDR pathogen | 0 | 0.0 | 7 | 100.0 | 0.191 |
| Study center |  |  |  |  |  |
| Center 1 | 5 | 31.3 | 11 | 68.8 | 0.179 |
| Center 2 | 14 | 27.5 | 37 | 72.5 |  |
| Center 3 | 0 | 0.0 | 4 | 100.0 |  |
| Center 4 | 1 | 8.3 | 11 | 91.7 |  |
| Center 5 | 8 | 12.3 | 57 | 87.7 |  |
| Center 6 | 18 | 19.6 | 74 | 80.4 |  |
| Median total duration of antibiotic treatment [p25;p75] | 10 [7;12] | | 11 [9;14] | | 0.013 |

a. Denominators varied due to missing values. Total numbers for respective variables were: sex (n=46, n=192), Charlson Comorbidity Index (n=46, n=190), ICU admission in the last 3 months (n=44, n=182), Pitt bacteremia score day 3 (n=46, n=177)

Abbreviations: BSI – bloodstream infection, ICU – intensive care unit, MDR – multidrug resistance

# **S Table 9: Most used antibiotics before switch among those with IVOS, full list (n=240)**

| **Antibiotic** | **n** | **%** |
| --- | --- | --- |
| Piperacillin/tazobactam | 60 | 25.0 |
| Ampicillin/sulbactam | 48 | 20.0 |
| Ceftriaxone | 32 | 13.3 |
| Meropenem | 25 | 10.4 |
| Cefotaxime | 22 | 9.2 |
| Ciprofloxacin | 21 | 8.8 |
| Ampicillin | 4 | 1.7 |
| Cefuroxime | 4 | 1.7 |
| Ampicillin/sulbactam + clarithromycin | 2 | 0.8 |
| Piperacillin/tazobactam + ciprofloxacin | 2 | 0.8 |
| Piperacillin/tazobactam + clarithromycin | 2 | 0.8 |
| Piperacillin/tazobactam + linezolid | 2 | 0.8 |

# **S Table 10: Most used antibiotic classes before switch among those with IVOS (n=240)**

| **Antibiotic** | **n** | **%** |
| --- | --- | --- |
| Ureidopenicillin/beta-lactamase inhibitor | 60 | 25.0 |
| Third-generation cephalosporin | 54 | 22.5 |
| Aminopenicillin/beta-lactamase inhibitor | 48 | 20.0 |
| Carbapenem | 25 | 10.4 |
| Fluoroquinolone | 22 | 9.2 |
| Aminopenicillin | 9 | 3.8 |
| Second-generation cephalosporin | 4 | 1.7 |
| Ureidopenicillin/beta-lactamase inhibitor + fluoroquinolone | 4 | 1.7 |
| Third-generation cephalosporin + fluoroquinolone | 2 | 0.8 |
| Aminopenicillin/beta-lactamase inhibitor + macrolide | 2 | 0.8 |
| Ureidopenicillin/beta-lactamase inhibitor + linezolid | 2 | 0.8 |
| Ureidopenicillin/beta-lactamase inhibitor + macrolide | 2 | 0.8 |

# **S Table 11: Most used antibiotics after switch among those with IVOS, full list (n=240)**

| **Antibiotic** | **n** | **%** |
| --- | --- | --- |
| Ciprofloxacin | 75 | 31.3 |
| Sultamicillin | 56 | 23.3 |
| Cefuroxime axetil | 27 | 11.3 |
| Cefpodoxime | 22 | 9.2 |
| Amoxicillin/clavulanic acid | 18 | 7.5 |
| Cotrimoxazole | 11 | 4.6 |
| Levofloxacin | 10 | 4.2 |
| Amoxicillin | 5 | 2.1 |
| Linezolid | 5 | 2.1 |
| Moxifloxacin | 3 | 1.3 |

# **S Table 12: Most used antibiotic classes after switch among those with IVOS (n=240)**

| **Antibiotic** | **n** | **%** |
| --- | --- | --- |
| Fluoroquinolones | 88 | 36.7 |
| Aminopenicillin/beta-lactamase inhibitor | 74 | 30.8 |
| Second-generation cephalosporin | 27 | 11.3 |
| Third-generation cephalosporin | 24 | 10.0 |
| Trimethoprim/sulfamethoxazole | 11 | 4.6 |
| Aminopenicillin | 6 | 2.56 |
| Linezolid | 5 | 2.1 |
| Macrolides | 1 | 0.4 |
| Quinolone + linezolid | 1 | 0.4 |
| Quinolone + aminopenicillin | 1 | 0.4 |

# **S Table 13: Most used empiric antibiotics among those with an option for IVOS (n=742)**

| **Antibiotic** | **n** | **%** |
| --- | --- | --- |
| Piperacillin/tazobactam | 212 | 28.6 |
| Ampicillin/sulbactam | 121 | 16.3 |
| Ceftriaxone | 121 | 16.3 |
| Meropenem | 57 | 7.7 |
| Cefotaxime | 29 | 3.9 |
| Ciprofloxacin | 28 | 3.8 |
| Ampicillin/sulbactam + clarithromycin | 9 | 1.2 |
| Cefuroxime | 9 | 1.2 |
| Piperacillin/tazobactam + ciprofloxacin | 9 | 1.2 |
| Piperacillin/tazobactam + meropenem | 7 | 0.9 |

# **S Table 14: Most used day 7 antibiotics among those with an option for IVOS (n=742)**

| **Antibiotic** | **n** | **%** |
| --- | --- | --- |
| Piperacillin/tazobactam | 122 | 16.4 |
| Ampicillin/sulbactam and sultamicillin | 117 | 17.8 |
| Ceftriaxone | 91 | 12.3 |
| Ciprofloxacin | 86 | 11.6 |
| Meropenem | 78 | 10.5 |
| Cefuroxime | 30 | 4.0 |
| Cefpodoxime | 23 | 3.1 |
| Amoxicillin/clavulanic acid | 20 | 2.7 |
| Ampicillin | 18 | 2.4 |
| Cefotaxime | 15 | 2.0 |

# **S Figure 1: Most frequently used antibiotics and change in their use in patients switched to oral therapy by day 7 (n=240)**

Carbapenem + glycopeptide

Carbapenem + linezolid

2nd gen cephalosporin + aminopenicillin

DAY 7

Linezolid

Aminopenicillin

Carbapenems

Other

3rd gen cephalosporin

Cotrimoxazole

2nd gen cephalosporin

Aminopenicillin/beta-lactamase-inhibitor

Quinolone

DAY 3

2nd gen cephalosporin

Other

Quinolone

Aminopenicillin/beta-lactamase-inhibitor

Piperacillin/

tazobactam

3rd gen cephalosporin

# **S Figure 2: Most frequently used antibiotics and change in their use in patients not switched to oral therapy by day 7 (n=502)**

2nd gen cephalosporin

Piperacillin/tazobactam + glycopeptide

Linezolid

Carbapenem + linezolid

Carbapenem + glycopeptides

Aminoglycoside

Carbapenem + glycopeptides

Piperacillin/tazobactam + quinolone

3rd gen cephalosporin + linezolid

Linezolid

Carbapenem + linezolid

DAY 7

DAY 3

Aminopenicillins

Other

Quinolones

Quinolones

Other

Aminopenicillin/beta-lactamase-inhibitor

Aminopenicillin/beta-lactamase-inhibitor

Carbapenem

Carbapenem

3rd gen cephalosporin

3rd gen cephalosporin

Piperacillin/

tazobactam

Piperacillin/

tazobactam

# **S Table 15: Final multivariable mixed effect logistic regression model of factors associated with 28-day mortality (n=644^a^)**

| **Variables** | **Multivariable Final Model** | |
| --- | --- | --- |
|  | **Adjusted odds ratio (OR) with 95% CI** | **p-value** |
| Cancer diagnosis |  |  |
| No | 1 |  |
| Yes | 1.85 (1.03-3.30) | **0.038** |
| Metastasized solid tumor |  |  |
| No | 1 |  |
| Yes | 2.14 (1.13-4.07) | **0.020** |
| Pitt Bacteremia Score day 3 |  |  |
| < 2 | 1 |  |
| ≥ 2 | 2.65 (1.58-4.44) | **<0.001** |
| Focus of infection |  |  |
| All other foci | 1 |  |
| Urogenital focus | 0.45 (0.22-0.94) | **0.032** |
| Cardiovascular focus | 0.30 (0.09 – 1.00) | 0.050 |
| Site of acquisition |  |  |
| Community-acquired | 1 |  |
| Healthcare-associated | 1.70 (0.97-2.97) | 0.061 |
| Hospital-acquired | 2.53 (0.93-6.90) | 0.070 |
| Switch to oral therapy |  |  |
| No | 1 |  |
| Yes | 0.05 (0.01-0.28) | **<0.001** |

a. Data available for all the variables included in the final model for 644 of the 742 patients.

Variables included in the full multivariable model: Gram stain of infecting pathogen, Cancer diagnosis, Metastasized solid tumor, Focus of infection, Dementia, Moderate-to-severe liver disease, Pitt Bacteremia Score day 3, Charlson Comorbidity index, Site of acquisition, Acute kidney injury before day 7, Switch to oral therapy.
